# Healthcare systems and health economics in GCC countries: informing decision-makers from the perspective of the Gulf health economics association

**DOI:** 10.3389/fpubh.2025.1510401

**Published:** 2025-03-25

**Authors:** Yazed AlRuthia, Sara Aldallal, Hana A. Al-Abdulkarim, Ahmed Al-jedai, Hajer Almudaiheem, Anas Hamad, Khalifa Elmusharaf, Mouza Saadi, Hamda Al Awar, Haleama Al Sabbah, Suliman Alghnam, Mohamed Al Ghamdi, Sarah S. Monshi, Nada AlAgil, Mohamed Ebrahim Al Khalifa, Qasim Abdulkarim, Sawsan Abdulkarim, Huda Jawad, Sultana Al-Sabahi, Asiya Al Kindi, Said Wani, Abdullah Alibrahim

**Affiliations:** ^1^Pharmacoeconomics Research Unit, Department of Clinical Pharmacy, College of Pharmacy, King Saud University, Riyadh, Saudi Arabia; ^2^Dubai Health Authority, Dubai, United Arab Emirates; ^3^Drug Policy and Economic Center, National Guard Health Affairs, Riyadh, Saudi Arabia; ^4^King Abdullah International Medical Research Center, King Saud Bin Abdulaziz University for Health Sciences, Riyadh, Saudi Arabia; ^5^Therapeutics Affairs, Ministry of Health, Riyadh, Saudi Arabia; ^6^College of Pharmacy and Medicine, Alfaisal University, Riyadh, Saudi Arabia; ^7^Department of Drug Policy and Regulation, Ministry of Health, Riyadh, Saudi Arabia; ^8^Department of Pharmacy, National Center for Cancer Care and Research, Hamad Medical Corporation, Doha, Qatar; ^9^College of Pharmacy, QU Health Sector, Qatar University, Doha, Qatar; ^10^Public Health Programme at the University of Birmingham, Dubai, United Arab Emirates; ^11^Department of Health Economics and Insurance Policies, Dubai Health Authority, Dubai, United Arab Emirates; ^12^Department of Health, Data Management and Healthcare Studies, Abu Dhabi, United Arab Emirates; ^13^Department of Public Health Nutrition, Abu Dhabi University, Abu Dhabi, United Arab Emirates; ^14^Saudi Public Health Authority, Riyadh, Saudi Arabia; ^15^General Directorate of National Health Process and Economics, Saudi Health Council, Riyadh, Saudi Arabia; ^16^Department of Health Administration and Hospitals, College of Public Health and Health Informatics, Umm Al Qura University, Makkah, Saudi Arabia; ^17^Department of Medical, Council of Cooperative Health Insurance, Riyadh, Saudi Arabia; ^18^Health Strategic Assessment Directorate, Supreme Council of Health, Manama, Bahrain; ^19^Freelancer Management Consultant and Co-founder of the Bahrain Research Consortium, Manama, Bahrain; ^20^Department of Pharmacy, University of Bahrain, Manama, Bahrain; ^21^Ministry of Health, Muscat, Oman; ^22^Assistant Professor, Industrial and Management Systems Engineering, Kuwait University, Kuwait, Kuwait; ^23^Ministry of Health, Kuwait, Kuwait

**Keywords:** GCC (Bahrain; Saudi Arabia; Kuwait; United Arab Emirates; Qatar; Oman; Arabian Gulf; Persian Gulf), health technology assesement (HTA), healthcare system, healthcare finance, healthcare efficiency

## Abstract

Following the discovery of oil, citizens of the Cooperation Council for the Arab States of the Gulf (GCC), which includes Bahrain, Kuwait, Qatar, Oman, the United Arab Emirates, and Saudi Arabia, have enjoyed the benefits of universal healthcare. However, as the population and healthcare demands in the GCC continue to grow, financing these healthcare systems without adequately considering the value of reimbursed health technologies and the effectiveness of various policies has become increasingly challenging. While numerous narrative reviews and government reports have discussed the healthcare systems in these nations, they have not sufficiently addressed the approval processes, economic evaluations, and reimbursement mechanisms for health technologies. In response to this gap, experts in health economics and outcomes research (HEOR) from the Gulf Health Economics Association – recognized as key opinion leaders in public health and academia across the six GCC countries – conducted a focus group discussion. This focus group meeting, which was recorded and transcribed verbatim to be later thematically analyzed, aimed to characterize the current state of healthcare systems within the GCC, identify challenges in adopting and implementing health economic evaluations to inform policymakers and propose recommendations to expedite the integration of HEOR in the assessment of various health policies and technologies within their respective countries. The convened experts also underscored the importance of collaboration among GCC member states to enhance the adoption of robust health technology evaluations and improve patient access to cost-effective treatments.

## 1 Introduction

### 1.1 An overview of the healthcare systems in the GCC countries

Healthcare expenditures as a share of the Gross Domestic Product (GDP) varies among the GCC countries ranging from 2.6% in Qatar, 3.5% in the United Arab Emirates (UAE), 4.5% in the Kingdom of Bahrain, 5.3% in Kuwait, 5.7% in Saudi Arabia, and 6% in Oman based on the WHO Global Health Expenditure Database (1). However, experts believe that these figures may be conservative and can be much higher than what has been reported by the governments and the WHO, especially in the public healthcare systems, which are undergoing a comprehensive overhaul to improve quality, access, and efficiency. For example, the Saudi government annual budget allocation for the Ministry of Health has increased from 2.8% in 1970 to 8% in 2022 (2). All GCC citizens have universal healthcare coverage, which allows them to access public healthcare facilities with minimal to no out-of-pocket costs. Private healthcare insurance can be purchased for access to private facilities, and in some GCC countries, private sector employees are provided with healthcare coverage by their employers as required by law. The Kingdom of Saudi Arabia has taken steps to privatize its public healthcare sector, with various governmental and semi-governmental bodies now offering private health insurance for citizens and foreigners (3). Furthermore, Saudi Arabia has launched a comprehensive national health transformation program to privatize its public healthcare sector (4). Similarly, in the United Arab Emirates (UAE), nationals and non-nationals working in the private sector are covered with employer-sponsored private health insurance (5). In Kuwait, citizens working in the private sector are covered by public healthcare institutions, while retired individuals receive supplemental private health insurance that covers healthcare services up to 15,000 Kuwaiti Dinar ($49,114.31) (6), but this supplemental health insurance for retirees was suspended recently (7). Despite the universal healthcare coverage in GCC countries, significant out-of-pocket expenditures for essential healthcare services are reported in the GCC countries (1, 8, 9). The latest published figures on healthcare out-of-pocket expenditure as a percentage of the overall healthcare expenditure by the World Health Organization Global Health Expenditure Database in the GCC countries were in 2019, and were as follows: 16% in Saudi Arabia, 30% in Bahrain, 12% in Kuwait, 6.6% in Oman, 12% in Qatar, and 13% in the United Arab Emirates (10).

In the GCC countries, foreigners working in the public sector can receive healthcare from public healthcare institutions through different financing schemes (11). However, the situation is different for foreigners working in the private sector. In Bahrain, private-sector employers must provide healthcare coverage for their foreign employees. This mandate is fulfilled through compulsory health insurance, purchased from the Ministry of Health and managed by the Labor Market Regulatory Authority (LMRA). The annual cost for this basic insurance is approximately 72 Bahraini dinars (US$191) per employee. While this coverage includes primary care, outpatient prescription medications, and emergency services, some employers supplement it with additional private insurance or directly pay for private healthcare services (12). In Oman, non-citizens who are not working in the government sector are not covered by the public healthcare sector, and they need to obtain private health insurance since private employers are not required to provide it. Nonetheless, foreigners can access public healthcare institutions and pay for healthcare services based on a price list of medical services issued by the Omani Ministry of Health (13). In addition, new legislation is already in place and will soon make it mandatory for private employers to provide health insurance coverage in Oman. In Kuwait, a new public–private partnership health insurance scheme known as Dhaman, is preparing to commence operations and replace the previously discussed assurance model. Under this public–private partnership health insurance scheme, government and private employers would sponsor their non-Kuwaiti employees to be insured and receive care at Dhaman Health Assurance Hospitals Company. Premiums are currently set to be 130 Kuwaiti Dinars ($425.66) per employee. There is a fixed co-payment ranging from 5 Kuwaiti Dinars ($16.37) to 10 Kuwaiti Dinars ($32.74) for every visit, depending on the type of medical service received (12). In Saudi Arabia, private employers must provide all employees and their dependents with private health insurance covering basic health services approved by the Council of Health Insurance (CHI) (14, 15). In the UAE, citizens and foreigners have mandatory private health insurance in Dubai and Abu Dhabi. In the emirates of Ajman, Fujairah, Ras Al Khaimah, Sharjah, and Umm Al Quwain, there is currently no legal requirement for employers to provide medical insurance, whether public or private, to their employees. However, following a recent announcement by the UAE cabinet on March 18th, 2024, it will become mandatory for private-sector and domestic workers across all of the UAE, including the aforementioned emirates, to have health insurance starting January 1st, 2025. As a result, all private sector employers will be obliged to ensure that their employees are covered by health insurance (12). In Qatar, non-nationals have access to non-emergency healthcare services through the national healthcare system funded by the government. They pay out-of-pocket nominal fees to access different healthcare services. Efforts are underway to make the employer-sponsored healthcare insurance mandatory in the foreseeable future for those employed in the private sector (12).

Although the public healthcare systems in the GCC countries are similar in many aspects, some differences should be highlighted (16). In Saudi Arabia, the public healthcare system is fragmented. Citizens and their dependents working in the civil government ministries receive medical care through the Ministry of Health hospitals and primary care clinics. Those with rare and complex medical conditions, such as cancer and rare diseases, can be referred to advanced public health institutions, such as King Faisal Specialist Hospital and Research Center (2). Civil and military employees and their dependents in the Ministries of National Guard, Defence, and Interior receive medical care in the hospitals and primary care clinics affiliated with these ministries (2). In addition, employees working in academic institutions receive care from hospitals and primary care clinics affiliated with these academic organizations (2). This fragmentation in the public healthcare system results in poor coordination between different public health organizations, lack of a centralized electronic medical record that captures the patient journey in different public healthcare organizations, and duplication of medical services (2). In the other GCC countries, the public health systems are fragmented but to a much lesser degree than in Saudi Arabia, and medical services are primarily provided through hospitals and primary care clinics affiliated with the Ministry of Health and Military hospitals for those working in the military (1, 11, 16). Similarly, there are issues with healthcare integration between different hospitals and data completeness in electronic medical records. However, Qatar and the United Arab Emirates have more integrated healthcare systems, particularly Dubai and Abu Dhabi (1, 2).

The financing of healthcare systems in the GCC countries relies mainly on the export revenues from natural resources (oil and gas). Therefore, the public expenditures on healthcare represent 77% of the healthcare expenditures in Saudi Arabia, 66% in Bahrain, 64.1% in the United Arab Emirates, 90% in Kuwait, 88% in Oman, and 85% in Qatar (2). Besides the fragmented healthcare systems, the GCC countries face numerous challenges regarding quality, access, and efficiency in healthcare management, escalating healthcare costs, demographic changes, lower fertility rates, aging populations, and increasing incidence rates of chronic and genetic diseases (17). Additionally, the share of public spending on ambulatory care services and preventive care in Saudi Arabia, for example, does not exceed 18 and 3%, respectively, and more than 78% of the public spending on healthcare goes to hospital services (2). The share of public expenditure on hospital services in the Organization for Economic Cooperation and Development (OECD) countries does not exceed 39% (18). The situation in other GCC countries (Kuwait, Bahrain, United Arab Emirates, Qatar, and Oman) does not differ much from Saudi Arabia; however, no official estimates have been published yet in these countries (17).

## 2 Knowledge gaps and objectives

Although the healthcare systems in the GCC countries have been described in multiple narrative reviews and published government reports, these studies or reports have yet to characterize the approval, economic evaluations, and reimbursement of health technologies in these countries. Therefore, the paper aims to collect insights about the current status of health technology approval and reimbursement, and identify the challenges in conducting health economic evaluations through a focused group meeting with key opinion leaders in the public health sector and academic institutions from the six GCC countries.

## 3 Methods

In order to evaluate the current state of health technology approvals and economic assessments, as well as to explore the potential establishment of national health technology assessment (HTA) bodies and their future roles in the reimbursement of health technologies, we conducted a qualitative focus group study. This study brought together 21 key opinion leaders from the six GCC countries. The selection of experts was based on the following criteria:

1. They hold an advisory role in a large public healthcare system in their respective countries, specifically related to the evaluation and procurement of health technologies.
2. They occupy a senior position within a regulatory body, such as the Ministry of Health or a local health authority.
3. They are affiliated with a reputable academic institution in their country and have a proven track record of research publications on public health and health technology assessment, in addition to serving as consultants to various regulatory bodies in their respective countries.

The participants represented Ministries of Health and healthcare systems (10 participants), prominent academic institutions (four participants), and regulatory bodies (seven participants). A meeting guide consisting of 16 open-ended questions was developed following a comprehensive literature review to characterize health systems in the GCC countries. This guide comprised of three main themes:

1. Healthcare Delivery Systems in the GCC Countries.
  - Public vs. Private Sector.
  - Access and Equity.
  - Funding Sources.
  - Insurance Models.
2. Disease Epidemiology in the GCC Countries.
  - Prevalent Chronic Health Conditions.
  - Access to Therapy.
  - Barriers to Access.
  - Gaps in Preventive Care.
3. HTA in Healthcare Decision-Making in the GCC Countries.
  - It’s Role in Informing Policy and Resource Allocation.
  - Data Availability and Quality.
  - Challenges in Implementing HTA.

This guide aimed to describe the healthcare systems and their challenges, including the structure of the healthcare systems in the GCC countries and the most prevalent health conditions. It also aimed to describe the current landscape of health technology approval and economic evaluation and discusses the future role of health economic evaluation in informing decision-makers about the value and reimbursement of various health technologies in the GCC countries. During the focus group discussions, which were moderated by the senior author [YA], verbal and non-verbal communication cues were observed and documented by the first two authors [YA and SD]. They also recorded and transcribed the meeting verbatim. The transcripts underwent inductive thematic analysis, with key excerpts extracted manually by the first and second authors (19). The first and second authors [YA and SD], who possess considerable experience in qualitative research, subsequently reviewed these themes, which were manually extracted based on frequency before being shared with the other authors. The data were managed in accordance with the ethical principles outlined in the Helsinki Declaration, ensuring that all responses were anonymized (20).

The four-hour closed-door meeting took place in Dubai on June 28, 2024. Representatives from each of the six GCC countries met at separate round tables to facilitate dialogue and ensure efficient time management. The first author served as the moderator for the session, which was video recorded and transcribed verbatim to capture a range of themes. All participants have agreed to join this meeting and record the session. The Gulf Health Economics Association organized and conducted the meeting, and no ethical approval was required.

## 4 Results and discussion

The key opinion leaders at the meeting discussed various topics summarized under the following themes that were validated by different published official reports and studies:

### 4.1 The approval and reimbursement of health technologies in the GCC countries

In the GCC countries, the registration and approval of health technologies, including medications and medical devices, are typically overseen by the respective Ministries of Health. However, Bahrain and Saudi Arabia have established independent regulatory authorities for this purpose. The Bahrain National Health Regulatory Authority (NHRA) is responsible for regulating health technologies in Bahrain, while the Saudi Food and Drug Authority (SFDA) fulfils this role in Saudi Arabia (21, 22). Pricing for pharmaceuticals and medical devices is primarily determined through external reference pricing from a selection of reference countries and the country of origin (21, 22). The proportion of healthcare spending allocated to pharmaceuticals varies across the GCC countries, ranging from 11% in Qatar to 21.7% in Oman (22, 23). However, some healthcare experts argue that these statistics underestimate the actual spending on medical goods, such as pharmaceuticals and medical devices, and suggest that it could be as high as 30% of total health spending in the UAE (9, 24, 25).

In the GCC countries, pharmaceuticals and medical devices in the public health systems are reimbursed by the Ministry of Health and other relevant public healthcare institutions (e.g., military hospitals, medical cities, etc.…) (26). The procurement process varies in each country. In Saudi Arabia, public healthcare institutions are mandated by law to obtain medical devices and medications through the National Unified Procurement Company (NUPCO) using centralized tenders (27). Private healthcare institutions in Saudi Arabia can procure medications and medical devices through their tenders or by negotiating directly with manufacturers or wholesalers, purchasing pharmaceuticals and medical devices registered by the SFDA (28). In the United Arab Emirates, citizens pay for healthcare services and medications through the National Health Insurance Company (Thiqa and Daman) (9). There is no single procuring body for the procurement of pharmaceuticals and medical devices in the UAE. For example, in Abu Dhabi, the Abu Dhabi Developmental Holding Company (ADQ) has established RAFED, a Group Purchasing Organization (GPO) responsible for the procurement needs of Abu Dhabi Health Services Company (SEHA) at local and regional levels (9, 29). In Oman, Qatar, Bahrain, and Kuwait, public healthcare entities secure their medications and medical devices through centralized tenders for public health institutions. Additionally, these countries, along with Saudi Arabia and the UAE, participate in the Gulf Joint Procurement Program to procure some public health institutions’ medications, vaccines, and medical devices (30). Private health institutions and insurance plans in Kuwait, Oman, Bahrain, and Qatar procure medications and medical devices directly from local or international markets.

### 4.2 Prevailing health conditions and health indicators in GCC countries

The Gulf Cooperation Council (GCC) countries are facing significant challenges related to non-communicable diseases (NCDs) such as diabetes, hypertension, dyslipidemia, ischemic heart disease, stroke, obesity, and cancer (1, 31). The prevalence rates of diabetes in GCC countries far exceed the global prevalence rate, with figures of 18.3% in Saudi Arabia, 15.4% in the United Arab Emirates, 22% in Kuwait, 15.7% in Oman, 15.5% in Qatar, and 16.3% in Bahrain (1, 32, 33). Similarly, the prevalence rates of obesity in the GCC region are considerably higher than the global average, with an average prevalence rate of 23.7% compared to the global rate of 12.91% (11, 34). The economic burden of NCDs in the GCC countries is substantial, with an estimated cost of illness associated with four major NCDs (cancer, diabetes, cardiovascular diseases, and chronic respiratory diseases) reaching USD 50 billion in 2019, representing about 3.3% of the GCC GDP (35). Furthermore, consanguineous marriages are prevalent in the GCC countries, resulting in high rates of genetic disorders such as thalassemia and sickle cell disease (SCD) (36). For instance, the prevalence rates of SCD in Saudi Arabia, Bahrain, and Qatar were estimated to be 3.3, 12, and 2.05%, respectively (37). While efforts to address genetic disorders through preventative measures such as pre-marital screening and genetic counseling have been implemented in the GCC countries, the success rates vary (38–40). Notably, the United Arab Emirates has reported relatively lower prevalence rates of *β*-thalassemia and SCD compared to other GCC countries due to effective preventative measures (37, 41). Additionally, other serious genetic conditions, including Duchenne Muscular Dystrophy and Spinal Muscular Atrophy, have shown increasing incidence rates in the past decade (42–44).

Although the GCC countries have made significant leaps toward improving the health status of their citizens, there is still a long way to go. The average life expectancy at birth in 2022 was estimated to be 74 years, 78 years, 79 years, 80 years, and 82 years, in Oman, Saudi Arabia, Bahrain and United Arab Emirates, Kuwait, and Qatar, respectively. Although this is higher than the global average life expectancy of 72 years, with the exception of Qatar, this is below the average life expectancy in some OECD countries with lower GDP PPP per capita, such as the United Kingdom (82 years), France (82 years), and Canada (81 years) (45). Moreover, the infant mortality rates per 1,000 live births is 10 in Oman, 8 in Kuwait, 6 in Saudi Arabia and Bahrain, and 5 in Qatar and United Arab Emirates, which are higher than the ones reported for France and the United Kingdom (4 per 1,000 live births) (45). Additionally, the maternal mortality ratios per 100,000 live births in Oman was 17, and 16 in Bahrain and Saudi Arabia, which are significantly higher than the ones reported in the United Arab Emirates (9 per 100,000 live births), Qatar (8 per 100,000 live births), and Kuwait (7 per 100,000 live births), but lower than the world average of 223 per 100,000 live births (45).

### 4.3 The role of health technology assessment in informing healthcare decision-making in the GCC countries

#### 4.3.1 The status-quo of health economic evaluations of different policies and technologies in the GCC countries

The field of health economics and outcomes research (HEOR) is still evolving in the Arabian GCC countries. Before any medication is registered and priced by the SFDA, a thorough review of cost-effectiveness studies is conducted to advise decision-makers on the value of new therapies compared to other registered medications with similar labeled indications. However, the review of cost-effectiveness studies and different health technology assessment agencies, such as the National Institute for Health and Care Excellence (NICE) and the Canadian Agency for Drugs and Technologies in Health (CADTH), does not replace the central role of external reference pricing for all newly registered pharmaceuticals (46). Starting from July 2025, economic evaluations will be required by the SFDA for all submitted drug registration applications (47). However, medical devices do not undergo similar rigorous evaluation.

After a medication is registered by the SFDA, various healthcare institutions such as the Ministry of Health, National Guard Health Affairs, Ministry of Defense Health Affairs, King Faisal Specialist Hospital and Research Center, and others evaluate the newly approved medications upon request by their prescribers for inclusion in the formulary. The evaluation primarily focuses on the clinical effectiveness of the newly approved medications compared to other medications with similar labeled indications. Budget impact analysis is requested by multiple healthcare institutions, mainly the Ministry of Health Therapeutic Affairs, and efforts are ongoing to make the submission of cost-effectiveness analysis a requirement in the future. Moreover, the Ministry of Health Therapeutic Affairs has published guidance for the financial and outcome-based Managed Entry Agreements in 2021 (48) and has signed multiple outcome-based agreements over the past four years, which are now in effect (49). Furthermore, the newly established Health Technology Assessment Center will inform the decision-making process regarding the adoption and reimbursement of different health technologies (i.e., medications and medical devices) for the public healthcare institutions under the Ministry of Health (50, 51). Additionally, tentative cost-effectiveness thresholds and EQ-5D-5L value set have recently been established for use in future health economic evaluations (52, 53). Moreover, the Council of Health Insurance (CHI) requires budget impact analysis and cost-effectiveness evaluation for any prescription medication before inclusion in the Insurance Drug Formulary, in addition to a thorough clinical review (54). Furthermore, the role of non-profit organizations such as the Saudi Society of Pharmacoeconomics and Outcomes Research and the Saudi chapter of the International Society of Health Economics and Outcomes Research (ISPOR) in shaping policies and informing health decision-makers is increasing.

In the United Arab Emirates (UAE), budget impact analyses are carried out for certain new therapies prior to their inclusion in the formulary for various insurance plans in Dubai and Abu Dhabi. However, budget impact analyses for new therapies are rarely requested in the Northern Emirates (Ras Al Khaimah, Sharjah, Fujairah, Umm Al Quwain, and Ajman) where insurance is not mandatory and the public healthcare system provides coverage for citizens. Cost-effectiveness or cost-utility analyses are not mandated for the reimbursement of any pharmaceuticals or medical devices in all emirates. Nonetheless, efforts are being made to establish utility value sets, and a pilot program for a multi-criteria decision analysis (MCDA) tool was initiated for the procurement of generic pharmaceuticals (55). Moreover, the Emirates Society of Health Economics is playing an increasing vital role in improving awareness about the value of health economic evaluation in informing decision-makers and improving spending efficiency.

In Oman and Bahrain, prior to inclusion in the public health system formulary, medications undergo budget impact analyses. Additionally, the central drug committee is regularly informed about the incremental value of any new medication through a review of published cost-effectiveness studies. However, reimbursement for health technology in Oman, Kuwait, Qatar, and Bahrain does not require economic evaluations for medications or medical devices, despite the growing research output in health economics and outcomes research (HEOR) in Qatar (29).

#### 4.3.2 Challenges in conducting health economic evaluation in the GCC countries

Over the past decade, there has been significant progress in adopting and implementing health economic evaluation across the GCC countries. However, several shared challenges still exist and must be addressed, these can be summarized as follows:

1. Limited awareness and support from stakeholders to include health economic evaluation as a requirement for reimbursing any health technology.

There is currently a limited understanding and lack of comprehensive support among key stakeholders regarding the inclusion of health economic evaluations as a critical criterion for the reimbursement of health technologies in the Gulf Cooperation Council (GCC) countries, including Saudi Arabia, Qatar, Kuwait, the United Arab Emirates, Oman, and Bahrain. This gap in awareness may hinder the effective assessment of cost-effectiveness and overall impact on healthcare systems, resulting in suboptimal allocation of resources and potentially delaying patient access to innovative treatments. Fostering greater recognition and support for health economic evaluations is essential to ensure that reimbursement decisions are informed, transparent, and aligned with the broader goals of improving healthcare outcomes in the region.

2. Fragmented healthcare systems and different reimbursement schemes.

The healthcare systems in the Gulf Cooperation Council (GCC) countries are characterized by fragmentation and a lack of coordination. This often leads to discrepancies in care delivery, with various sectors operating independently rather than in a cohesive manner. As a result, patients may face challenges such as inconsistent quality of services, limited access to comprehensive care, and difficulties navigating the different regulatory frameworks across the member states. The absence of integrated health policies and communication among healthcare providers further exacerbates these issues, ultimately impacting the overall health outcomes for the population in the region.

3. Issues in healthcare data quality, ownership, and governance.

The healthcare sector in the GCC countries encounters significant challenges related to data quality, ownership, and governance. These issues manifest in various ways, adversely affecting patient care, data management, and the overall efficiency of healthcare systems. The accuracy, completeness, and timeliness of healthcare data are essential for effective decision-making and positive patient outcomes. However, inconsistencies in data collection methods and varying standards across different healthcare institutions often lead to fragmented and unreliable information. This lack of data integrity hampers comprehensive analyses, impacting everything from patient diagnoses to resource allocation, a concern commonly observed in GCC healthcare systems. Moreover, determining data ownership within the healthcare settings of GCC countries is complex. Clear definitions of data ownership are crucial to ensuring legal compliance and safeguarding patient privacy. Additionally, there is frequently a lack of standardized governance policies in the region, which can result in inconsistent practices among institutions. This absence of cohesion may lead to data security vulnerabilities and potential breaches of patient confidentiality. Therefore, it is vital to establish comprehensive governance structures that prioritize accountability and transparency to enhance trust in the healthcare system.

4. Varied perceptions and perspectives among different stakeholders.

Various stakeholders within the GCC healthcare systems often possess diverse perceptions and viewpoints regarding health economic evaluations. These differing perspectives can arise from factors such as professional roles, personal experiences, and the specific interests of each group. For instance, policymakers may prioritize cost-effectiveness to ensure the optimal allocation of financial resources, whereas healthcare providers might concentrate on patient outcomes and the quality of care. In contrast, patients and their advocates often emphasize the accessibility and affordability of treatments. This diversity in viewpoints can lead to different interpretations of data and findings, resulting in difficulty in shaping a unified policy within each GCC healthcare system.

5. Insufficient resources in terms of human expertise, data coding, and capacity to conduct health economic evaluation.

There is a significant shortage of skilled professionals with expertise in health economics across the GCC countries. This gap is compounded by a lack of individuals who are proficient in data coding and analysis, which is essential for conducting effective health economic evaluations. Moreover, the infrastructure and resources necessary to support comprehensive health economic assessments are insufficient. This shortfall hinders the capacity to make informed healthcare decisions and restricts the potential for improving health policies and resource allocations in the region. Addressing these challenges is crucial for enhancing the overall health system and ensuring more effective healthcare delivery in the GCC.

6. Lack of national cost-effectiveness thresholds in all GCC countries except Saudi Arabia.

The GCC countries face a pressing challenge when conducting cost-effectiveness analysis due to the absence of established national cost-effectiveness thresholds, except Saudi Arabia. This lack of formal benchmarks leaves nations like the United Arab Emirates, Qatar, Kuwait, Oman, and Bahrain without essential guidelines for evaluating the cost-effectiveness of vital healthcare interventions and treatments. Consequently, this gap hinders informed decision-making in healthcare policy and resource allocation, ultimately impacting the quality of care. In contrast, Saudi Arabia has proactively developed its cost-effectiveness thresholds, creating a solid framework for evaluating healthcare expenditures. This enhances decision-making and paves the way for more sustainable healthcare financing strategies across the kingdom.

7. Absence of utility value sets and costing data in most GCC countries.

The lack of comprehensive utility value sets and detailed costing data in the majority of GCC countries, with the notable exceptions of Saudi Arabia and the United Arab Emirates, poses significant challenges in carrying out health economic evaluations. This absence hinders the ability to accurately assess the cost-effectiveness of healthcare interventions, leading to difficulties in developing evidence-based health policies. Without standardized utility values and reliable cost data, researchers and policymakers find it challenging to compare health outcomes and make informed decisions regarding resource allocation and investment in healthcare services across the region.

8. Legislative challenges, including constitutional rights surrounding citizen access to therapy and willingness to pay in some GCC countries such as Kuwait.

Legislative challenges surround the constitutional rights related to citizens’ access to therapy and willingness to pay. In all GCC countries, access to healthcare is recognized as a right rather than a privilege. However, the situation becomes complicated when access to innovative health technologies comes at a high cost. Patients across the GCC countries face delays in receiving care that relies on these expensive health technologies, mainly when the evidence regarding their cost-effectiveness is uncertain. Additionally, healthcare payers encounter legal challenges when rationing costly health technologies or applying eligibility criteria based on economic considerations. This is primarily due to constitutional rights that guarantee citizens access to healthcare without any conditions or specifications, such as utilizing health economic evaluations to determine whether patients will be granted certain medications or medical devices, especially in Kuwait.

9. Limited research grants to support health economics and outcomes research.

Opportunities for funding research in health economics and outcomes within the GCC countries remain limited. However, countries such as Saudi Arabia and the United Arab Emirates increasingly recognize the importance of research investment. They are committing substantial financial resources to hundreds of millions of dollars towards various research initiatives. These efforts predominantly emphasize basic science, technological innovation, and clinical research. Unfortunately, this focus often overlooks crucial areas like health economics and outcomes research (HEOR), which are essential for understanding healthcare interventions’ economic impacts and improving patient outcomes. As the region continues to develop its healthcare systems and seeks to enhance the efficiency and effectiveness of healthcare delivery, a stronger emphasis on HEOR could provide valuable insights that support informed policymaking and resource allocation decisions.

10. Lack of institutionalization of health economic evaluation within healthcare evaluation processes.

It is notable that there is no systematic and formal approach to incorporating health economic evaluations into the healthcare assessment frameworks within Gulf Cooperation Council (GCC) countries. This lack of institutionalization hinders the effective integration of economic considerations into healthcare decision-making processes, which could ultimately impact resource allocation, cost-effectiveness, and the overall efficiency of healthcare delivery in the region. Without established guidelines and structures for health economic evaluations, stakeholders may struggle to make informed decisions that balance clinical outcomes with financial sustainability.

11. Suboptimal coordination between academia and decision-makers.

There is a significant gap in collaboration between academic institutions and healthcare decision-makers in the GCC countries regarding integrating health economic evaluations into the healthcare decision-making process. This lack of effective coordination hampers the ability to utilize evidence-based findings that could inform policies and resource allocation in the healthcare sector. As a result, there is an ongoing need for improved dialogue, partnerships, and frameworks that facilitate the exchange of knowledge and research between these two crucial sectors, ultimately promoting more effective and sustainable healthcare solutions in the region.

12. Lack of formal agreements to collaborate between different GCC countries that protect the rights of participating institutions in health economic evaluation projects.

There is a notable deficiency in formal agreements that promote collaboration among the Gulf Cooperation Council (GCC) countries, particularly regarding protecting the rights and responsibilities of institutions involved in health technology assessment projects. This absence of structured agreements hinders effective communication, coordination, and resource sharing, compromising the region’s quality and efficiency of health technology evaluations. Without clear guidelines and mutual protections, participating institutions may encounter challenges related to intellectual property, data sharing, and the equitable distribution of benefits arising from joint assessments. The extracted themes regarding the challenges in adopting and implementing HTA in the GCC countries are shown in Table 1.

**Table 1:** Qualitative themes and sub-themes for the challenges in adopting and implementing HTA in the GCC countries.

Theme	Subthemes	Quotes
Limited awareness and support	Lack of stakeholder understanding. Insufficient support for health economic evaluations. Barriers to integration in reimbursement processes. Impact on resource allocation and patient access. Need for increased recognition and advocacy. Importance of informed and transparent reimbursement decisions. Alignment with healthcare outcome improvement goals.	“In ……policy-makers are aware of the importance of economic evaluation. However, their level of awareness is limited to the financial aspect of any policy or coverage expansion and not to the cost-effectiveness of specific health policies or technology. Therefore, we do not have the resources to conduct such comprehensive evaluations….”
Fragmented healthcare systems	Inconsistent policy frameworks. Different perspectives among decision-makers. Variable reimbursement schemes and quality of care.	“We lack a coordinated healthcare system. Some patients can access multiple public healthcare facilities, leading to the repetition of the same procedures within a short time. Also, some patients fill their prescription medications at various hospitals.…”
Data issues	Inconsistency in data collection and validation. Ambiguity in data ownership. Lack of standardized governance.	“Some public hospitals have good data quality, mainly from their electronic health records, but many do not since they do not use the same metrics.”
Capacity and resources	Human resource limitations Financial incentives Infrastructure and technological support	“In…. We have an HTA unit within the Ministry of Health, but we have very few staff and still lack the resources, such as cost data, to conduct a full health economic evaluation.”
Absence of cost-effectiveness thresholds	Informed decision-making dilemmas. Resource allocation challenges.	“We do not have a cost-effectiveness threshold because we do not conduct full health economic evaluation in….”
Research and development	Research priorities in GCC Academic contributions and publications Establishment of research centers	“The research priorities and funding opportunities in the GCC countries are different. We need to streamline this!”
Absence of costing data and utility value sets	Inconsistent HTA practices. Lack of well-defined decision-making framework. Dilemmas on health technology adoption. Reliance on HTA reports issued by foreign HTA bodies.	“In certain cases, BIA (Budget impact analysis) is required for reimbursement, while in others, it is conducted only at the academic level. Sometimes, this analysis can influence the decision-making process. This influence arises mainly from our lack of healthcare cost data, a defined cost-effectiveness threshold, and an established utility value set.”
Miscoordination between academia and decision-makers	Insufficient engagement strategies. Misalignment of research priorities. Need for structured partnerships. Investment in capacity building.	“In …… much of the HEOR research is conducted at academic institutions, but, its potential to drive impactful healthcare decision-making is still minimal.”
Absence of formal agreements for collaboration in HEOR between GCC countries	Legal and regulatory challenges. Data sharing and confidentiality concerns. Institutional relationships and trust.	“To my knowledge, there is no formal agreement between the GCC countries to share healthcare data for health economic evaluations, and we need it!”

## 5 Strengths and limitations

This study represents the first thorough and comprehensive review, to the best of our knowledge, of the healthcare systems within the Gulf Cooperation Council (GCC) countries, along with the multifaceted challenges associated with conducting health technology assessments at the national level. This exploration is informed by the insights and perspectives of esteemed key opinion leaders from each of the member nations, who provided valuable information reflecting the complexities and nuances of their respective healthcare landscapes. However, it is important to acknowledge certain limitations inherent to this study. One primary concern is the relatively small sample size, which may not adequately capture the diverse viewpoints across the region. Furthermore, the study was based on a single focus group discussion, which could limit the depth and breadth of dialogue surrounding these critical issues. Additionally, some key regulatory bodies, such as the Saudi Food and Drug Authority, were not included in the discussions, potentially leading to a less comprehensive understanding of the regulatory environment and the specific challenges faced by these organizations in the health technology assessment process.

## 6 Conclusion

To promote Health Economics and Outcomes Research (HEOR) collaboration among institutions in the GCC countries, it is crucial to engage decision-makers. Our expert panel has outlined steps for fostering collaboration, including identifying common research priorities by mapping research interests across healthcare and academic institutions in the Arabian GCC countries. The shared priorities include healthcare financing, sustainability of care models, cost-effectiveness of reimbursement schemes, defining value in care from the perspective of GCC populations, public-private partnerships, efficacy of public health policies, and cost-effectiveness of health technologies for managing chronic diseases. Additionally, the panel recommends establishing regional guidelines for health economic evaluations, identifying accessible healthcare data with acceptable quality, and setting rules and regulations to protect the rights of participating organizations. Furthermore, the establishment of a Gulf Health Economics Research Center within the Gulf Health Council is proposed.

## Data Availability

The original contributions presented in the study are included in the article/supplementary material, further inquiries can be directed to the corresponding author/s.

## References

[1] Fadhil I Ali R Al-Raisi SS Bin Belaila BA Galadari S Javed A. Review of National Healthcare Systems in the Gulf cooperation council countries for noncommunicable diseases management. Oman Med J. (2022) 37:e370. doi: 10.5001/omj.2021.96, PMID: 35602320 PMC9087205

[2] Nair KS Mughal YH Albejaidi F Alharbi AH. Healthcare financing in Saudi Arabia: a comprehensive review. Healthcare. (2024) 12:2544. doi: 10.3390/healthcare12242544, PMID: 39765971 PMC11675727

[3] Alasiri AA Mohammed V. Healthcare transformation in Saudi Arabia: an overview since the launch of vision 2030. Health Services Insights. (2022) 15:11786329221121214. doi: 10.1177/11786329221121214, PMID: 36081830 PMC9445529

[4] Rahman R. The privatization of health care system in Saudi Arabia. Health Services Insights. (2020) 13:1178632920934497. doi: 10.1177/1178632920934497, PMID: 32636636 PMC7315664

[5] Koornneef E Robben P Blair I. Progress and outcomes of health systems reform in the United Arab Emirates: a systematic review. BMC Health Serv Res. (2017) 17:672. doi: 10.1186/s12913-017-2597-1, PMID: 28931388 PMC5607589

[6] Mossialos E Cheatley J Reka H Alsabah A Patel N. Kuwait: Health system review. London School of Economics and Political Sciences. London, UK: LSE Health, (2018).

[7] Saha Suspends Supplemental Private Insurance Coverage for Retirees. (2024). *Aljarida*. Available online at: https://www.aljarida.com/article/74681 (Accessed October 21, 2024).

[8] Al-Hanawi MK. Decomposition of inequalities in out-of-pocket health expenditure burden in Saudi Arabia. Soc Sci Med. (2021) 286:114322. doi: 10.1016/j.socscimed.2021.114322, PMID: 34454127

[9] Hassan R Sher HA Khokhar R Hussain R. Pharmaceutical policy in the UAE. Pharm Policy Countries Developing Healthcare Systems. (2017):365–79. doi: 10.1007/978-3-319-51673-8_18

[10] WHO. (2019). *Global Health Expenditure Database*. Available online at: https://www.undp.org/arab-states/publications/costing-primary-healthcare-arrangements-six-gulf-cooperation-council-countries-synthesis-report

[11] Khoja T Rawaf S Qidwai W Rawaf D Nanji K Hamad A. Health Care in Gulf Cooperation Council Countries: a review of challenges and opportunities. Cureus. (2017) 9:e1586. doi: 10.7759/cureus.1586, PMID: 29062618 PMC5650259

[12] ILO. (2023). *Review of National Social Protection Legislation and legal frameworks for migrant Workers in the Gulf Countries (International Labour Organization)*. Available online at: https://www.ilo.org/sites/default/files/wcmsp5/groups/public/@arabstates/@ro-beirut/documents/publication/wcms_886063.pdf (Accessed October 12, 2024).

[13] Habersky E Damir A. COVID-19 financing strategies for refugees and migrants in the eastern Mediterranean region. East Mediterr Health J. (2021) 27:1229–38. doi: 10.26719/emhj.21.061, PMID: 35137391

[14] AlJohani BA Bugis BA. Advantages and challenges of implementation and strategies for health insurance in Saudi Arabia: a systemic review. Inquiry. (2024) 61:3447. doi: 10.1177/00469580241233447, PMID: 38357867 PMC10874142

[15] Council of Health Insurance. CHI’s enhanced essential benefit package: Prioritizing your Health & Wellbeing. Riyadh: Council of Health Insurance (2022).

[16] Al Mawali AHN Al Qasmi AM Al Sabahi SMS Idikula J Elaty MAA Morsi M. Oman Vision 2050 for Health Research: a strategic plan for the future based on the past and present experience. Oman Med J. (2017) 32:86–96. doi: 10.5001/omj.2017.18, PMID: 28439378 PMC5397084

[17] Alsubahi N Pavlova M Alzahrani AA Ahmad A e Groot W. Healthcare quality from the perspective of patients in gulf cooperation council countries: a systematic literature review. Healthcare. (2024) 12:315. doi: 10.3390/healthcare12030315 38338200 PMC10855039

[18] Kacholi G Anasel MG. Health at a glance 2023: OECD indicators. Paris: OECD Publishing. (2023).

[19] Terry G Hayfield N Clarke V Braun V. Thematic analysis. SAGE Handbook Qualitative Research Psychol. (2017) 2:25. doi: 10.4135/9781526405555.n2, PMID: 34907622

[20] Shrestha B Dunn L. The declaration of Helsinki on medical research involving human subjects: a review of seventh revision (2019) 17:548–52. doi: 10.33314/jnhrc.v17i4.1042, 32001865

[21] Howard JJ. Medical devices and the Middle East: Market, regulation, and reimbursement in gulf cooperation council states. Med Dev. (2014):385–95. doi: 10.2147/MDER.S73079 PMC4242697 25429243

[22] Kanavos P Tzouma V Fontrier A-M Kamphuis B Parkin GC Saleh S. Pharmaceutical pricing and reimbursement in the Middle East and North Africa region. London: LSE Consulting, London School of Economics and Political Science (2018).

[23] Abdel Rida N Mohamed Ibrahim MI Babar Z-U-D. Pharmaceutical pricing policies in Qatar and Lebanon: narrative review and document analysis. J Pharm Health Serv Res. (2019) 10:277–87. doi: 10.1111/jphs.12304 PMC6921405 31852546

[24] Alnaqbi KA Elshamy AM Gebran N Fahmy S Aldallal S Korra N. Consensus-based recommendations for the implementation of health technology assessment in the United Arab Emirates. Value Health Regional Issues. (2024) 43:101012. doi: 10.1016/j.vhri.2024.101012, PMID: 38861786

[25] Hasan RSI Lessing C. Pharmaceutical pricing policies in the Gulf countries’ council (GCC) and the United Arab Emirates (UAE) In: Pharmaceutical prices in the 21st century. Gewerbestrasse, Cham, Switzerland: Springer (2014). 297–307.

[26] Hamad A Alsaqa'aby M Alruthia Y Aldallal S Elsisi GH. Overview of procurement and reimbursement of Pharmaceuticals in Saudi Arabia, United Arab Emirates, Qatar, and Egypt: challenges and opportunities. Glob J Qual Saf Healthc. (2023) 6:127–36. doi: 10.36401/jqsh-23-1, PMID: 38404458 PMC10887475

[27] AlRuthia Y Alrashed SA Balkhi B Aljamal MS Alsifri S Alrumaih AM. COVID-19 and Saudi Arabia public financing of prescription drugs: an opportunity for reform. Health Policy Technol. (2021) 10:3–6. doi: 10.1016/j.hlpt.2020.10.008

[28] Alghaith T Almoteiry K Alamri A Alluhidan M Alharf A Al-Hammad B. Strengthening the pharmaceutical system in the Kingdom of Saudi Arabia. Washington, DC: World Bank. (2020).

[29] Hamad A Elazzazy S Bujassoum S Rasul K Gaziev J Cherif H. Applying value-based strategies to accelerate access to novel cancer medications: guidance from the oncology health economics expert panel in Qatar (Q-OHEP). BMC Health Serv Res. (2023) 23:15. doi: 10.1186/s12913-022-08981-5, PMID: 36609388 PMC9816531

[30] DeRoeck D Bawazir SA Carrasco P Kaddar M Brooks A Fitzsimmons J. Regional group purchasing of vaccines: review of the Pan American health organization EPI revolving fund and the Gulf cooperation council group purchasing program. Int J Health Plann Manag. (2006) 21:23–43. doi: 10.1002/hpm.822, PMID: 16604847

[31] Mohammed EM. High number of familial breast Cancer cases in the Arabian Gulf countries: investigating the reasons. Breast Cancer. (2022) 16:11782234221107121. doi: 10.1177/11782234221107121, PMID: 35783595 PMC9243472

[32] Al-Mawali A Al-Harrasi A Jayapal SK Morsi M Pinto AD Al-Shekaili W. Prevalence and risk factors of diabetes in a large community-based study in the Sultanate of Oman: STEPS survey 2017. BMC Endocr Disord. (2021) 21:42. doi: 10.1186/s12902-020-00655-9, PMID: 33673840 PMC7934365

[33] Aljulifi MZ. Prevalence and reasons of increased type 2 diabetes in gulf cooperation council countries. Saudi Med J. (2021) 42:481–90. doi: 10.15537/smj.2021.42.5.20200676, PMID: 33896777 PMC9149705

[34] El-Sahli Z. Globalization and obesity in the GCC countries. Middle East Dev J. (2023) 15:26–49. doi: 10.1080/17938120.2022.2160182

[35] Elmusharaf K Grafton D Jung JS Roberts E Al-Farsi Y Al Nooh AA. The case for investing in the prevention and control of non-communicable diseases in the six countries of the Gulf cooperation council: an economic evaluation. BMJ Glob Health. (2022) 7:e008670. doi: 10.1136/bmjgh-2022-008670, PMID: 35649631 PMC9161070

[36] Bener A Hussain R Teebi AS. Consanguineous marriages and their effects on common adult diseases: studies from an endogamous population. Med Princ Pract. (2007) 16:262–7. doi: 10.1159/000102147, PMID: 17541290

[37] Abu-Shaheen A Heena H Nofal A Abdelmoety DA Almatary A Alsheef M. Epidemiology of thalassemia in gulf cooperation council countries: a systematic review. Biomed Res Int. (2020) 2020:1509501. doi: 10.1155/2020/1509501, PMID: 33178817 PMC7644312

[38] Al Eissa MM Almsned F Alkharji RR Aldossary YM AlQurashi R Hawsa EA. The perception of genetic diseases and premarital screening tests in the central region of Saudi Arabia. BMC Public Health. (2024) 24:1556. doi: 10.1186/s12889-024-19029-0, PMID: 38858722 PMC11165879

[39] Memish ZA Saeedi MY. Six-year outcome of the national premarital screening and genetic counseling program for sickle cell disease and β-thalassemia in Saudi Arabia. Ann Saudi Med. (2011) 31:229–35. doi: 10.4103/0256-4947.81527, PMID: 21623050 PMC3119961

[40] Saffi M Howard N. Exploring the effectiveness of mandatory premarital screening and genetic counselling Programmes for *β-*Thalassaemia in the Middle East: a scoping review. Public Health Genomics. (2015) 18:193–203. doi: 10.1159/000430837, PMID: 26045079

[41] Natarajan J Joseph MA. Premarital screening for genetic blood disorders — an integrated review on the knowledge and attitudes of middle eastern university students. Middle East Fertility Soc J. (2021) 26:19. doi: 10.1186/s43043-021-00065-4

[42] Al Jumah M Al Rajeh S Eyaid W Al-Jedai A Al Mudaiheem H Al Shehri A. Spinal muscular atrophy carrier frequency in Saudi Arabia. Mol Genet Genomic Med. (2022) 10:e2049. doi: 10.1002/mgg3.2049, PMID: 36062320 PMC9651606

[43] Aldharee H. Duchenne muscular dystrophy in Saudi Arabia: a review of the current literature. Front Neurol. (2024) 15:1392274. doi: 10.3389/fneur.2024.1392274, PMID: 39087004 PMC11288836

[44] Rabea F El Naofal M Chekroun I Khalaf M Zaabi NA AlZaabi K. Spinal muscular atrophy genetic epidemiology and the case for premarital genomic screening in Arab populations. Commun Med. (2024) 4:119. doi: 10.1038/s43856-024-00548-1, PMID: 38879606 PMC11180197

[45] WHO. (2022). The Global Health Observatory. Available online at: https://www.who.int/data/gho. (Accessed October 9, 2024).

[46] Khan TM Emeka P Suleiman AK Alnutafy FS Aljadhey H. Pharmaceutical pricing policies and procedures in Saudi Arabia: a narrative review. Ther Innov Regul Sci. (2016) 50:236–40. doi: 10.1177/2168479015609648, PMID: 30227009

[47] SFDA. Economic Evaluation Studies Guidelines. Riyadh, Saudi Arabia: Saudi and Drug Authority (2024).

[48] Saudi MOH. Managed entry agreement policy for Saudi MOH. Riyadh, Saudi Arabia: Saudi Ministry of Health (2021).

[49] Abu-Shraie N Alhammad A Balkhi B Al-Jedai A. Implementation of risk-sharing agreements in Saudi Arabia: comparison and reflection on the NICE model. Trop J Pharm Res. (2023) 22:1121–31. doi: 10.4314/tjpr.v22i5.27

[50] Al-Aqeel S. Health technology assessment in Saudi Arabia. Expert Rev Pharmacoecon Outcomes Res. (2018) 18:393–402. doi: 10.1080/14737167.2018.1474102, PMID: 29733227

[51] Al-Omar HA Attuwaijri AA Aljuffali IA. What local experts expect from a health technology assessment (HTA) entity in Saudi Arabia: workshop conclusions. Expert Rev Pharmacoecon Outcomes Res. (2020) 20:99–104. doi: 10.1080/14737167.2019.1610398, PMID: 31032687

[52] Al-Jedai A Almudaiheem H Al-Salamah T Aldosari M Almutairi AR Almogbel Y. Valuation of EQ-5D-5L in the Kingdom of Saudi Arabia: a National Representative Study. Value Health. (2024) 27:552–61. doi: 10.1016/j.jval.2024.01.017, PMID: 38342365

[53] Al-Jedai AH Lomas J Almudaiheem HY Al-Ruthia YSH Alghamdi S Awad N. Informing a cost-effectiveness threshold for Saudi Arabia. J Med Econ. (2023) 26:128–38. doi: 10.1080/13696998.2022.2157141, PMID: 36576804

[54] Council of Health Insurance. Drug company external submission format. Riyadh, Saudi Arabia: Council of Health Insurance (2024).

[55] Farghaly MN Al Dallal SAM Fasseeh AN Monsef NA Suliman EAMA Tahoun MA. Recommendation for a pilot MCDA tool to support the value-based purchasing of generic medicines in the UAE. Front Pharmacol. (2021) 12:680737. doi: 10.3389/fphar.2021.680737, PMID: 34168564 PMC8217964

